# Dominant negative *Bmp5 *mutation reveals key role of BMPs in skeletal response to mechanical stimulation

**DOI:** 10.1186/1471-213X-8-35

**Published:** 2008-04-01

**Authors:** Andrew M Ho, Paul C Marker, Hairong Peng, Andres J Quintero, David M Kingsley, Johnny Huard

**Affiliations:** 1 Stem Cell Research Center, Children's Hospital of Pittsburgh, Pittsburgh, Pennsylvania 15213, USA; 2 Department of Genetics, Cell Biology, and Development, University of Minnesota Cancer Center, MMC 806, 420 Delaware St. SE, Minneapolis, Minnesota 55455, USA; 3 Department of Developmental Biology and Howard Hughes Medical Institute, Beckman Center B300, Stanford University School of Medicine, Stanford, California 94305, USA; 4 Departments of Molecular Genetics and Biochemistry and Bioengineering, University of Pittsburgh, Pittsburgh, Pennsylvania 15213, USA

## Abstract

**Background:**

Over a hundred years ago, Wolff originally observed that bone growth and remodeling are exquisitely sensitive to mechanical forces acting on the skeleton. Clinical studies have noted that the size and the strength of bone increase with weight bearing and muscular activity and decrease with bed rest and disuse. Although the processes of mechanotransduction and functional response of bone to mechanical strain have been extensively studied, the molecular signaling mechanisms that mediate the response of bone cells to mechanical stimulation remain unclear.

**Results:**

Here, we identify a novel germline mutation at the mouse *Bone morphogenetic protein 5 *(*Bmp5*) locus. Genetic analysis shows that the mutation occurs at a site encoding the proteolytic processing sequence of the BMP5 protein and blocks proper processing of BMP5. Anatomic studies reveal that this mutation affects the formation of multiple skeletal features including several muscle-induced skeletal sites *in vivo*. Biomechanical studies of osteoblasts from these anatomic sites show that the mutation inhibits the proper response of bone cells to mechanical stimulation.

**Conclusion:**

The results from these genetic, biochemical, and biomechanical studies suggest that BMPs are required not only for skeletal patterning during embryonic development, but also for bone response and remodeling to mechanical stimulation at specific anatomic sites in the skeleton.

## Background

An area of significant interest in orthopaedics and rehabilitation medicine is the effect of mechanical loading on bone formation and remodeling. Mechanical stimulation plays an important role in determining bone mass and density in the adult skeleton, as well as susceptibility to conditions such as fractures or osteoporosis. It has long been observed that bone mass and mineral density can be altered at very specific sites of the skeleton in response to mechanical stimulation during exercise, as seen in increased size and cortical thickness of the arm bone from the dominant side in tennis players [1-3] and the increased mineralization seen in the lumbar spine of weight lifters [4] or in the heel bone of runners [5]. In general, increased exercise or muscular loading will increase bone mass [6,7] or bone density [8,9]. In contrast, decreased loading will reduce osteogenic activity, as seen in the bones of test animals in space flight [10] or of patients in prolonged bed rest [11].

Since Wolff's observation in 1892 that mechanical stress is a primary determinant in bone adaptation [12], extensive studies have been performed to understand how bone responds to its mechanical environment. Frost proposed a "mechanostat" theory [13] in which the skeleton senses mechanical stimuli that are above a certain threshold and bone formation is activated. After cell-mediated bone remodeling, a feedback system resets this threshold. However, the exact mechanism by which this mechanostat converts biophysical force to a cellular response is unknown. Various mechanisms have been proposed to involve hydrostatic pressure [14,15], mechanical stretch [16-19], fluid shear [20-22], and others. The signals activated by these mechanisms have been postulated to act via mechanically sensitive ion channels [23-25], the integrin-cytoskeleton pathway [26-28], phospholipase C [17,29,30], or G protein cascades [31,32] to trigger a cellular response.

Bone morphogenetic proteins (BMPs) belong to the Transforming growth factor-beta (TGF-β) family of secreted signaling molecules [33]. Although previous studies have revealed much about the important role of BMPs in skeletal patterning in embryogenesis, many of these studies were limited by two issues. First, since BMPs are required for multiple aspects of organogenesis, loss of function mutations often produce animals with prenatal lethality due to pleiotropy [34-37]. Second, multiple coexpressing BMPs can produce functional redundancy and mask the effect of loss of function of a single BMP [38-40].

Previous null mutations identified at the *short ear/Bmp5 *locus have shown that early condensation and growth of cartilage precursors in the ear, rib, and vertebra require BMPs [41,42]. In 1987, a new *Bmp5 *mutation causing unusually short ears in mice arose spontaneously at The Jackson Laboratory. To gain further insight into the role of *Bmp5 *in skeletal development, these mice were used to identify the location of this novel *Bmp5 *mutation and its effect on the processing and activity of BMP5.

To further investigate the role of BMPs in development, mice which were homozygous for this novel *Bmp5 *mutation were generated. Our findings indicate that the mutation disrupts the processing of the BMP5 peptide and may inactivate BMP5. Furthermore, these mutant mice displayed severe defects at specific skeletal structures that were even more severe than those of *Bmp5 *null mutants. Some of the skeletal defects were observed at sites of bone-muscle interaction. Biomechanical studies show that mutant osteoblasts from these sites failed to respond appropriately to mechanical strain and may implicate BMPs as the endogenous signals for bone formation in response to mechanical stimulation.

## Results

### *Bmp5 *cleavage mutation disrupts the proteolytic processing of the BMP5 protein

**Figure 1:**
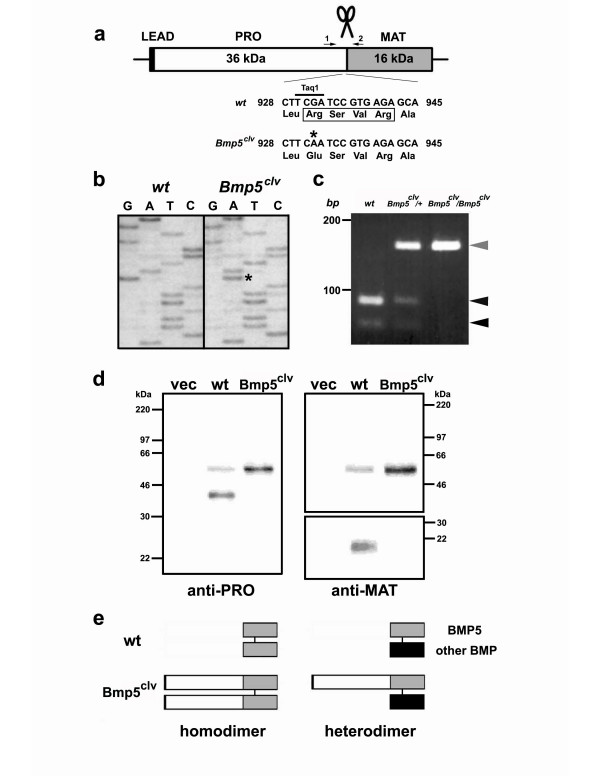
***Bmp5*^*clv*^, a *Bmp5 *cleavage sequence mutation, disrupts the proteolytic processing of the BMP5 protein**. **(a) **Schematic of the *Bmp5 *open reading frame showing the leader signal (LEAD), pro (PRO) and mature (MAT) domains and the putative proteolytic processing site (scissors). The G-to-A mutation (*) at nucleotide 932 of the *Bmp5*^*clv *^allele is predicted to destroy a Taq1 restriction site and to disrupt the first arginine residue in the putative conserved "RXXR" cleavage sequence in BMP5 (box). "1" and "2" denote positions of PCR primers used in typing the *Bmp5*^*clv *^allele. **(b) **Partial sequence traces showing the G-to-A substitution (*) in *Bmp5*^*clv *^mutant mice. This alteration is the only nucleotide difference in the *Bmp5 *coding region between wild-type (*wt*) and *Bmp5*^*clv *^mice. **(c) **Confirmation of mutation in genomic DNA. A 158-bp PCR product (gray arrowhead) containing the site of the *Bmp5*^*clv *^mutation is cleaved by Taq1 into 67- and 91-bp fragments (black arrowheads) in wild-type mice, partially cleaved in *Bmp5*^*clv*^/*+ *heterozygous mice, and not cleaved in *Bmp5*^*clv*^/*Bmp5*^*clv *^mice. **(d) **COS-7 cells were transfected with a mammalian expression vector (vec) or the same vector driving expression of wild-type (wt) or mutant (*Bmp5*^*clv*^) BMP5 protein. Secreted proteins were analyzed by Western blot with antibodies against the pro (anti-PRO) or mature (anti-MAT) domain of murine BMP5. Most of the wild-type protein expressed was in the smaller cleaved form, whereas all the detectable mutant protein was non-processed. No appreciable signal was detected by either antibody in the control cells. **(e) **A proposed dominant-negative mechanism for the *Bmp5*^*clv *^mutation. Wild-type BMP5 peptides are cleaved at the proteolytic site to form functional dimers with another wild-type copy of BMP5 (gray bar) or another related BMP (black bar). Cleavage mutants produce non-processed BMP5 peptides that bind other non-processed BMP5 peptides to form inactive homodimers or bind and sequester wild-type BMP5 or other related BMPs in defective heterodimers.

Sequencing of this newly discovered *Bmp5 *mutation revealed a correctly spliced *Bmp5 *transcript with a G-to-A substitution at base 932 of the *Bmp5 *coding region (Fig. 1a, b). This change destroys a Taq1 site in the second exon (Fig. 1c), providing a simple assay for following the mutation in genetic crosses.

Most TGF-β superfamily proteins are synthesized as larger precursors that are cleaved at an RXXR consensus site by proprotein convertase endoproteases to generate an N-terminal pro domain and a C-terminal signaling domain [43,44]. The G-to-A mutation changes the first arginine in the RXXR processing site of BMP5 to a glutamine (Fig. 1a). To test whether the Arg311Gln mutation disrupts BMP5 protein processing, we expressed the wild-type or mutant forms in COS-7 cells and analyzed conditioned media by Western blot analysis with antibodies raised to the pro and mature regions of BMP5. Cells transfected with the wild-type construct produced BMP5 protein bands of ~40 kDa and ~20 kDa, consistent with the expected sizes of the cleaved BMP5 pro and mature domains (Fig. 1d). Both antibodies also detected a minor protein band of ~55 kDa (Fig. 1d), suggesting that some secreted BMP5 protein was unprocessed. Cells expressing mutant BMP5 produced only the unprocessed ~55 kDa protein form (Fig. 1d), confirming that the mutation in the RXXR site blocks normal proteolytic cleavage. We termed this mutation *Bmp5*^*clv *^to denote the lesion at the cleavage site.

### *Bmp5*^*clv *^mutants exhibit an array of skeletal defects

Mutations in the RXXR site of other TGF-β family members have inhibited the processing and activity of the corresponding protein [45-48]. Such mutations also have acted as dominant-negative mutations that block the function of other coexpressed TGF-β members, presumably by sequestering them into inactive heterodimer complexes with the unprocessed mutant subunits [45,47]. Mice heterozygous for the *Bmp5*^*clv *^mutation showed mild skeletal defects not seen in wild-type or *+/Bmp5*^*null *^mice, including reduction of the spinous process at the second thoracic vertebrae (data not shown). Such defects are consistent with a mild dominant-negative effect observed when the *Bmp5*^*clv *^allele is present in single copy, consistent with the mode of action observed for similar mutations in other TGF-β members [45,47].

**Figure 2:**
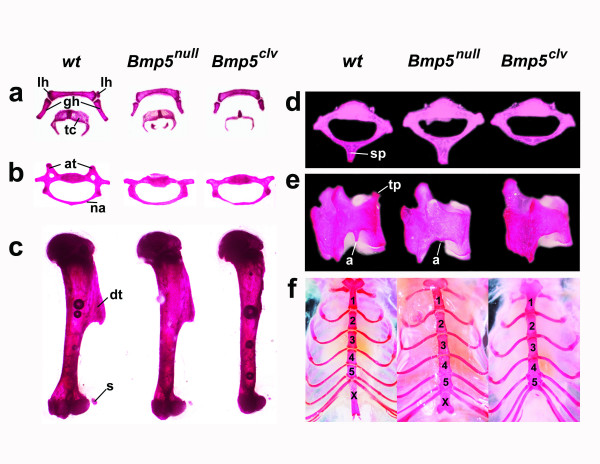
***Bmp5*^*clv *^mutation causes more severe skeletal defects than a *Bmp5 *null (*Bmp5*^*null*^) mutation**. Alizarin red-stained bones of 12-week +/+, *Bmp5*^*null*^/*Bmp5*^*null*^, and *Bmp5*^*clv*^/*Bmp5*^*clv *^mice show the following: **(a) **shortening of the greater horn (gh) and lesser horn (lh) of the hyoid bone and decreased calcification of the thyroid cartilage (tc), **(b) **absence of anterior tubercles (at) and thinning of the neural arch (na) of the 6th cervical vertebra, **(c) **reduction of the sesamoid (s) and nearly complete loss of the deltoid tuberosity (dt) of the humerus, **(d) **absence of the spinous process (sp) of the 2nd thoracic vertebra, **(e) **absence of the transverse process (tp) and anapophysis (a) of the 3rd lumbar vertebra, and **(f) **abnormal fusion of posterior sternal segments and loss of the xiphoid process (x) at the end of the sternum in *Bmp5*^*clv*^/*Bmp5*^*clv *^mice.

We expect homozygosity for the mutation to further decrease the activity of BMP5 and increase the production of the non-processed BMP5 molecules that may inactivate other coexpressed BMPs. To determine the effect of this mutation on homozygotes carrying this allele, we crossed heterozygous carriers of the *Bmp5*^*clv *^mutation and generated viable *Bmp5*^*clv*^/*Bmp5*^*clv *^homozygotes, but at rates ~10-times lower than Mendelian predictions (4/156 progeny, *P *< 0.001). Despite the increased prenatal lethality, some *Bmp5*^*clv *^homozygotes survived with normal life spans and fertility. Among these surviving homozygous *Bmp5*^*clv *^mice, we noted more severe defects than those seen with age-controlled *Bmp5*^*null *^homozygotes, including shorter external ears (wild-type: 6.4 ± 0.4 mm, null: 4.8 ± 0.2 mm, *Bmp5*^*clv*^: 2.9 ± 0.2 mm; *P *< 0.01), loss of lesser horns of the hyoid (Fig. 2a), more misshapen xiphisternum and missing ribs (Fig. 2f), less calcification of thyroid cartilage (Fig. 2a), abnormal bony fusion (fused posterior sternum; Fig. 2f), and reduced or absent processes on the sixth cervical (Fig. 2b), second thoracic (Fig. 2d), and lumbar vertebrae (Fig. 2e). The spectrum of phenotypes, and the consistent reduction rather than overgrowth of skeletal tissue, both suggest that the *Bmp5*^*clv*^mutation leads to loss rather than gain of BMP5 activity.

### *Bmp5*^*clv *^mutants display a deficiency at a mechanosensitive site of the skeleton

An interesting new phenotype in *Bmp5*^*clv *^mice is marked reduction or complete elimination of the deltoid crest on the humerus bone (Fig. 2c). The deltoid crest is a prominent bony ridge that is the insertion site for the deltoid muscle, and it normally forms in response to mechanical interaction between muscle and bone. In paralyzed animals or those exhibiting genetically defective muscle formation, the deltoid crest does not form [49,50]. In contrast, mutant animals showing abnormal increases in muscle mass develop bigger deltoid tuberosities [51].

**Figure 3:**
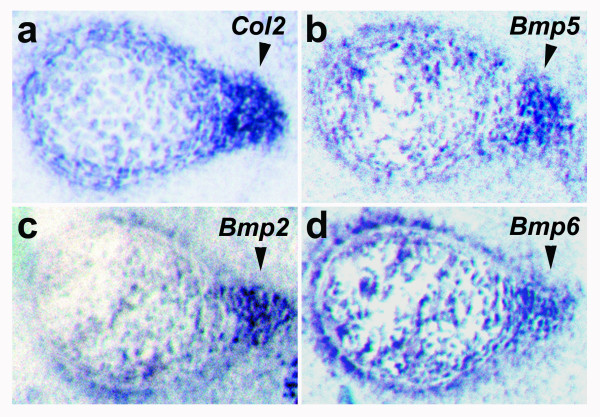
**Multiple *Bmps *are expressed at the deltoid tuberosity**. *In situ *hybridization analysis of the developing deltoid tuberosity at embryonic day 13.5 with antisense probes to **(a) ***collagen 2*, **(b) ***Bmp5*, **(c) ***Bmp2*, or **(d) ***Bmp6 *shows expression of multiple *Bmps *at the developing deltoid tuberosity (arrowheads). Control sense probes did not detect any appreciable signal (data not shown).

The deltoid muscle remained present in *Bmp5*^*clv*^*/Bmp5*^*clv *^mutants, suggesting that the deltoid crest defect was likely due to changes in the humerus bone. *In situ *hybridization showed *Bmp5 *expression in the developing deltoid crest of the humerus (Fig. 3b), and coexpression of the *Bmp2 *and *Bmp6 *genes in similar regions (Fig. 3c, d). The coexpression of multiple *Bmp*s may explain why the deltoid crest is only mildly reduced in *Bmp5*^*null*^mice but completely eliminated in *Bmp5*^*clv *^mice. The expression of multiple *Bmp *genes at the deltoid crest and the defect of this structure in *Bmp5*^*clv *^mutants suggest that *Bmp *signaling is important at this mechanosensitive site.

### *Bmp5*^*clv *^mutation alters the response of deltoid crest osteoblasts to mechanical stimulation

**Figure 4:**
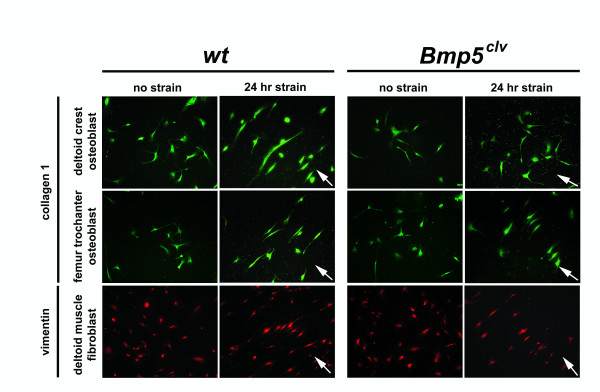
**Altered response to mechanical stimulation in deltoid tuberosity osteoblast cells of *Bmp5*^*clv*^/*Bmp5*^*clv *^mutants**. Cultured cells from the indicated sites were subjected to 0- or 24-h cyclic mechanical strain and visualized afterwards by indirect immunofluorescence using antibodies against collagen 1 (osteoblast) or vimentin (fibroblast). After subjection to 24-h strain, most osteoblasts from the deltoid tuberosity of wild-type (*wt*) mice become spindle-shaped and reorient perpendicular to the strain axis (arrow). In contrast, *Bmp5*^*clv *^deltoid tuberosity osteoblasts display a random orientation after mechanical strain. Femur trochanter osteoblasts from wild-type or *Bmp5*^*clv *^mice both realign after mechanical strain. Deltoid muscle fibroblasts from wild-type or *Bmp5*^*clv *^mice also respond appropriately to mechanical strain.

The loss of a prominent muscle-induced skeletal feature in *Bmp5*^*clv *^mice suggests that *Bmp *signaling plays a key role in bone cells' response to mechanical activity. To test this hypothesis, we isolated osteoblasts from the deltoid tuberosity of 10-month-old wild-type and *Bmp5*^*clv *^mice and subjected the cells to 24 hours of cyclic uniform radial strain in culture. The stretch regimen we applied (10-second maximum 15% elongation, then 10-second relaxation, frequency 0.05 Hz or 3 cycles/minute) is similar to the mechanical stimulation known to induce cellular responses in cultured osteoblasts [18,19]. After 24-hour cyclic strain, control osteoblasts became spindle-shaped, showed elongation of cellular processes, and were largely oriented perpendicular to the radial strain field (Fig. 4 and Table 1). In contrast, osteoblasts from the deltoid tuberosity of the *Bmp5*^*clv *^mice displayed no significant changes in morphology or orientation after mechanical strain (Fig. 4 and Table 1), suggesting that the defect in BMP signaling blocked normal response of bone cells to mechanical stimulation.

**Table 1:** Response of primary osteoblasts and muscle fibroblasts to cyclic mechanical strain.

**Cell type**	**[noggin](μg/mL)**	**Degree angle from strain axis**	**P value**
*wt *DT Os	-	82.3 ± 7.0 (91)	-
*Bmp5*^*clv *^DT Os	-	52.2 ± 24.5 (80)	<0.001
*wt *FT Os	-	80.9 ± 8.5 (95)	-
*Bmp5*^*clv *^FT Os	-	82.5 ± 6.5 (93)	NS
*wt *DM Fib	-	85.7 ± 3.8 (106)	-
*Bmp5*^*clv*^DM Fib	-	84.6 ± 4.7 (101)	NS
*wt *DT Os	0	83.5 ± 5.7 (123)	-
	0.1	80.2 ± 8.7 (148)	<0.01
	1	71.1 ± 16.7 (111)	<0.001
	10	62.3 ± 21.5 (107)	<0.001

**Cell type**	**Time strained (min)**	**% Nuclear SMAD**	**P value (*wt *vs. *Bmp5*^*clv*^)**

*wt *DT Os	0	7.1 ± 3.6 (94)	-
	10	15.4 ± 6.4 (99)	-
	20	34.2 ± 3.6 (139)	-
	30	35.8 ± 8.5 (131)	-
	60	32.4 ± 8.2 (124)	-
	24 h	8.6 ± 3.1 (146)	-
*Bmp5*^*clv *^DT Os	0	9.1 ± 4.6 (86)	NS
	10	13.7 ± 5.2 (94)	NS
	20	14.9 ± 5.5 (101)	<0.01
	30	18.4 ± 4.3 (100)	<0.05
	60	14.7 ± 1.6 (107)	<0.05
	24 h	7.5 ± 4.5 (90)	NS
*wt *FT Os	0	7.9 ± 6.3 (101)	-
	10	15.8 ± 8.6 (124)	-
	20	31.2 ± 3.3 (123)	-
	30	37.6 ± 1.3 (133)	-
	60	30.0 ± 7.3 (149)	-
	24 h	7.8 ± 4.3 (121)	-
*Bmp5*^*clv *^FT Os	0	8.4 ± 2.7 (101)	NS
	10	12.7 ± 7.9 (126)	NS
	20	34.5 ± 6.1 (134)	NS
	30	32.4 ± 5.0 (106)	NS
	60	26.4 ± 9.6 (111)	NS
	24 h	8.2 ± 3.0 (105)	NS

Values displayed are means ± SD of independent measurements from individual cells (cell # in parentheses) pooled from 3 experiments. p values between wild type and mutant data from the same cell type and time point are shown. *wt *= wild type, DT = deltoid tuberosity, FT = femur trochanter, DM = deltoid muscle, Os = osteoblast, Fib = fibroblast, NS = not significant.

Osteoblasts cultured from an independent bone-muscle interaction site that does not show morphologic defects in *Bmp5*^*clv *^mice responded normally to mechanical strain *in vitro *(femur trochanter osteoblasts; Fig. 4). The anatomic site-specificity of osteoblast response to mechanical stimuli in *Bmp5*^*clv *^mice is consistent with the specificity of the skeletal defects seen at the tissue level of these mutants. Furthermore, muscle fibroblasts isolated from the deltoid of both wild-type and *Bmp5*^*clv *^mice showed normal changes in morphology and orientation after mechanical stimulation *in vitro *(Fig. 4 and Table 1), suggesting that the mutation primarily affects bone cells and not muscle cells at these sites.

**Figure 5:**
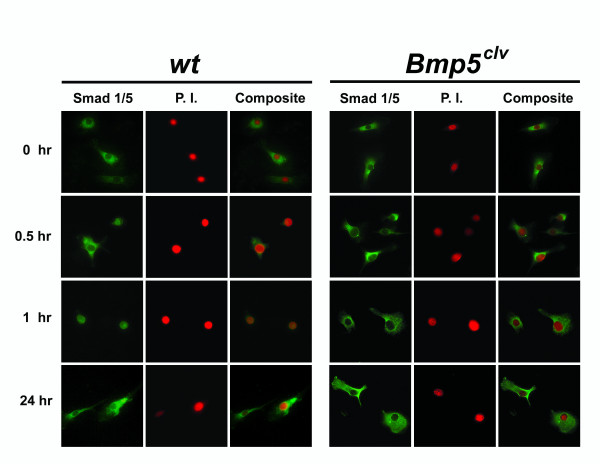
**SMAD1/5 nuclear relocalization after mechanical stimulation in deltoid tuberosity cells**. Resting wild-type (*wt*) deltoid tuberosity osteoblasts display SMAD1/5 immunoreactivity in the cytoplasm; however, with increased duration of mechanical strain SMAD immunoreactivity becomes more nuclear. At 24 hours, most of the reoriented cells again display cytoplasmic localization of SMAD 1/5. In contrast, cyclic strain failed to elicit any significant nuclear translocation of SMAD1/5 immunoreactivity in *Bmp5*^*clv *^deltoid tuberosity osteoblasts during the time period tested. Nuclei were counterstained with propidium iodide (P.I.).

To further characterize the relationship between mechanical stimulation and BMP signaling, we studied the effect of mechanical strain on cellular translocation of SMAD proteins, key transcription factors that translocate from the cytoplasm to the nucleus upon activation of BMP receptors [52]. Non-strained wild-type deltoid tuberosity osteoblasts exhibited SMAD1/5 immunoreactivity predominantly in the cytoplasm (Fig. 5 and Table 1). Within 30 minutes of applying cyclic strain to these cells, we detected a significant increase in nuclear localization of SMAD1/5 (Fig. 5 and Table 1). Peak nuclear localization occurred 1 hour after mechanical stimulation, SMAD1/5 was then predominantly cytoplasmic again by 24 hours (Fig. 5 and Table 1), at which point cells also had reoriented perpendicularly to the strain axis. In contrast, SMAD1/5 in *Bmp5*^*clv *^deltoid tuberosity cells remained mostly cytoplasmic before and after cyclic stretch (Fig. 5 and Table 1), demonstrating that perturbation of BMP signaling by the *Bmp5*^*clv *^mutation disrupts bone cells' rapid response to mechanical stretch.

While mutant osteoblasts from deltoid crest displayed altered response to mechanical stimuli, it was unclear whether this was due to an ongoing requirement for BMP-mediated signaling or abnormal cell development at the deltoid crest of *Bmp5*^*clv *^mice. To distinguish between these possibilities, we tested the effect of transiently inhibiting BMP signaling in wild-type osteoblasts cultured with increasing concentrations of noggin, a secreted protein that binds BMP and inhibits its activity [53]. Incubation of adult wild-type osteoblasts with noggin produced dose-dependent decreases in their reorientation response to mechanical stimulation (Table 1), confirming that BMP signaling is important in maintaining the normal mechanical responses of osteoblasts at postnatal stages.

## Discussion

Although the role of BMPs in the formation of cartilage, bone, and other tissues during embryonic development is well-established, studies of their functions during postnatal development are complicated by both the requirement of BMP signaling for many developmental events (pleiotropy) and the overlapping expression and roles of multiple BMPs at particular sites (partial functional redundancy). The spontaneous *Bmp5*^*clv*^allele is a missense mutation at a site that encodes the consensus cleavage sequence of BMP5 and blocks the post-translational processing of the BMP5 protein. Interestingly, this allele mimics a form of dominant-negative mutation that researchers have artificially induced in other TGF-β members and injected into frog or fish embryos [46,48] or expressed in transgenic animals [45,47] in attempts to inhibit endogenous activity. The processing-defective *Bmp5*^*clv *^allele identified here may be able to inactivate multiple BMPs but only at sites of endogenous *Bmp5 *gene expression, providing a unique tool to uncover new functions of *Bmp *genes during embryonic and postnatal development.

Skeletal analyses show that the *Bmp5*^*clv *^allele has a dominant negative effect. *Bmp5*^*clv *^heterozygote animals show skeletal defects not seen in *Bmp5*^*null *^heterozygotes, and *Bmp5*^*clv *^homozygote animals have a worse phenotype compared to that of *Bmp5*^*null *^mutants. Interestingly, studies on others TGF-β members have also described a dominant negative phenomenon displayed by similar cleavage sequence mutants. In those studies, the non-processed mutant proteins exert their effect on coexpressed wild type proteins by sequestering them into inactive complexes [45-48]. It remains to be shown whether BMP5^*clv *^heterodimerizes with BMP2 or BMP6, which are co-expressed at the deltoid crest. Inactivation of BMP2 and BMP6 by the mutant BMP5 protein may explain why the deltoid crest is only mildly reduced in *Bmp5*^*null *^mice but severely reduced in *Bmp5*^*clv *^mice.

While the skeletal analysis was performed in adult *Bmp5*^*clv *^mutants, given the expression of *Bmp5 *seen at the early embryonic stages of humeral development in our expression studies, it is possible that BMPs exert their effect on this structure at an early developmental stage. It will be interesting to perform histological analysis at various development time points to better characterize the phenotype of this mutation at different stages of development.

The skeletal defects we observed in the *Bmp5*^*clv *^mutants are localized to specific structures. Interestingly, previous studies show that the expression of *Bmp5 *is controlled by an array of cis-acting regulatory sequences that drive *Bmp5 *expression at highly specific anatomical locations in the skeleton [54,55]. Accordingly, the unique expression pattern of *Bmp5 *may account for the specificity of skeletal defects seen in the *Bmp5*^*clv *^mutants.

The deltoid tuberosity defect in *Bmp5*^*clv *^mice suggests that the formation of a prominent muscle-induced bony structure in the humerus requires BMP signaling. Our biomechanical studies confirm that BMP signaling is fundamental to the early response of bone cells to mechanical stimulation. While we have shown that osteoblasts respond to mechanical stimulation by increasing the nuclear translocation of SMAD1/5, others have also shown similar *in vitro *activation of BMP signaling with mechanical stimulation. Previous studies have shown elevated BMP mRNA levels in response to 6 hours of tensile stress applied to neonatal mouse calvaria [56] or cyclic stress applied to cultured chick chondrocytes [57]. Our results demonstrate SMAD translocation in normal osteoblasts *in vitro *within 30 minutes of mechanical stimulation and show that *Bmp5*^*clv *^cells fail to undergo SMAD translocation or reorientation in response to similar mechanical strain. The altered response to mechanical loading *in vitro *and the lack of muscle-induced features in *Bmp5*^*clv *^mice provide strong genetic evidence that BMP signaling is integral to the normal response of bone cells to mechanical strain. While our data show that this mutation has an impact on osteoblasts, it remains to be determined whether perturbation of BMP signaling has any effect on chondrocytes from the deltoid crest.

While mutant osteoblasts from deltoid crest displayed altered response to mechanical stimuli, it was unclear whether this was due to an ongoing requirement for BMP-mediated signaling or abnormal cell development at the deltoid crest of *Bmp5*^*clv *^mice. The altered response of wildtype deltoid crest osteoblasts to mechanical strain when the BMP signaling pathway was blocked with noggin suggests that BMPs are important in maintaining the ability of bone cells to respond to mechanical strain postnatally. It will be interesting to perform further biomechanical studies on *Bmp5*^*clv *^osteoblasts in the presence of exogenous BMP5 protein to ascertain whether the *Bmp5*^*clv *^mutation also affects the proper cell development of osteoblasts at the deltoid crest.

## Conclusion

Over the years, researchers have recognized that the mechanical response of bone strongly influences human health and disease. Weight-bearing exercise can increase bone mass and density and can reduce the risk of fracture in millions of persons with predisposing factors including osteoporosis. Numerous studies have provided a better understanding of the mechanical stimuli that bone cells detect, the signaling pathways that transduce mechanical signals to the cell, and the nature of the cellular response. This study on *Bmp5*^*clv *^mice shows that BMP signaling is an important part of this mechanotransduction system in bone. Further studies may elucidate how the BMP pathway interacts with other signaling pathways in this process. Modulation of BMP signaling through recombinant protein or gene therapy may enable clinicians to potentiate the benefits of weight-bearing activity to the skeleton or improve the treatment of bony diseases caused by prolonged lack of mechanical stimulation.

## Methods

### Mouse strains and skeletal preparation

The *Bmp5*^*clv *^mutation was originally designated "*se*-^4*J*^" when discovered as a spontaneous mutation in a backcross between C57Bl/6J and B6.Cg-*Otop1*^*tlt *^(for further strain details, see JAX stock number 001496). The *Bmp5*^*clv *^stock was maintained in the laboratory of DMK by intercrossing *Bmp5*^*clv*^*/+ *heterozygotes. Mutants were identified by the short ear phenotype, and their genotype was confirmed by molecular typing as described in this paper. The classical *se *mutation was also maintained on the C57Bl/6J background (Jackson Laboratory stock number 000056). Skeletons from age- and sex-matched mice from different genotypes were fixed in 95% ethanol, processed using a potassium hydroxide/alizarin red (Sigma) staining procedure [58], disarticulated, and analyzed.

### Identification of the *Bmp5*^*clv *^mutation

The *Bmp5 *open reading frame from total lung RNA of a 1-month-old C57Bl/6J male mouse and an age- and sex-matched *Bmp5*^*clv*^*/Bmp5*^*clv *^mutant mouse was amplified. Reverse transcription was carried out with Superscript RT (Gibco) using 1.5 μg of RNA and 2.5 μL of 20-μM reverse primer (5'-CGC GGA TCC CTA GTG GCA GCC ACA CGA-3') in a 20-μL reaction at 37°C for 1 h. Polymerase chain reaction (PCR) was performed with Amplitaq DNA polymerase (Perkin-Elmer) in a 50-μL reaction containing 1 μL each of 20-μM forward (5'-CGC GGA TCC ACC ATG CAT TGG ACT GTA TTT TTA C-3') and reverse (as above) primers and 1 μL of the RT reaction product from above. The PCR products were purified from a 1% regular agarose gel with the Gene-clean kit (Bio 101), digested with BamHI, and cloned into pBluescript II SK(+) (Stratagene). The *Bmp5 *cDNA insert on both strands from multiple clones of each genotype was sequenced with the Sequenase kit (United States Biochemicals) and primers that span the *Bmp5 *open reading frame (primer sequences available upon request).

The genomic region affected by the *Bmp5*^*clv *^mutation was amplified with primers 1 (5'-AAA TCT GCT GGT CTT GTG GG-3') and 2 (5'-GGG TCC TGA TGA GAG TTG GA-3') in a 50-μL PCR reaction containing 2.5 μL each of 20-μM primers 1 and 2 and 1.5 μL of genomic DNA. The amplified products were digested by TaqI and separated by electrophoresis on a 3% low melt agarose gel.

### Expression and detection of BMP5 protein

COS-7 cells were cultured in Dulbecco's modified Eagle's medium (DMEM) supplemented with 10% fetal bovine serum (FBS, Hyclone), 2-mM GlutaMax, 0.1-mM non-essential amino acids, penicillin G (100 U/mL), and streptomycin (100 μg/mL) at 37°C in 5% CO_2_/95% air and were passaged at confluency and maintained according to standard tissue culture practices. All tissue culture reagents were from Gibco except as indicated otherwise.

The *Bmp5 *open reading frame was amplified from wild-type or *Bmp5*^*clv *^homozygous mice as described above, subcloned into the mammalian expression vector pCDNA3 (Invitrogen), and sequenced to ensure that no mutations were introduced in the cloning steps. In transfection experiments, 2 × 10^6 ^COS-7 cells were plated on a 100-mm tissue culture dish (Corning) and transfected with 3.3 μg of DNA and 10 μL of lipofectamine (Gibco) per dish under reduced serum condition for 7 h (as instructed by the manufacturer). The transfected cells were allowed to recover in full medium for 5 h, at which point the normal medium was replaced with reduced serum medium (growth medium with 1% FBS, 3 mL/plate). After ~60 h, the conditioned media from 2 identically treated plates were collected and pooled, clarified by centrifugation, made 2% SDS by addition of a 10% SDS stock, boiled for 10 min, and aliquoted and stored at -20°C.

Recombinant protein containing murine BMP5 pro region (amino acids 1–310) fused with thioredoxin was expressed under the IPTG-inducible pET32 system in BL21 E. Coli bacterial cells as instructed by the manufacturer (Novagen). Briefly, 8 L transformed bacterial culture was grown to a density of OD600 ~0.5 and induced with 1-mM IPTG for 4 h at 37°C. Cells were collected by centrifugation and resuspended in Buffer A (6-M Guanidine HCl, 0.1-M NaH2PO4, 0.01-M Tris pH 8.0, 5 mL/g cell pellet weight), and the clarified supernatant was incubated with Ni-NTA nickel resin beads (Qiagen) for 1 h at room temperature (RT). The beads were washed with 10 volumes of Buffer A, 10 volumes of Buffer B (8-M urea, 0.1-M NaH2PO4, 0.01-M Tris pH 8.0), 10 volumes of Buffer C (8-M urea, 0.1-M NaH2PO4, 0.01-M Tris pH 6.3) with 5-mM imidazole, 10 volumes of Super Buffer C (Buffer C plus 250-mM NaCl, 0.5% Tween-20, 10-mM β-mercaptoethanol, 100-mM imidazole), and 2 washes of 10 volumes of Buffer C with 20-mM imidazole. Bound protein was eluted by boiling the beads in 2.5× Laemmli buffer. Polyclonal antibodies against the BMP5 pro domain (anti-PRO) were raised by immunizing rabbits with recombinant BMP5 pro region protein at the Berkeley Antibody Company. Monoclonal antibodies against the human BMP5 mature domain (anti-MAT) were provided by Genetics Institute, Inc., and were shown to cross-recognize the highly conserved murine BMP5 mature region.

For western blot analysis, 60 μL of the processed SDS samples were boiled for 5 min in reducing Laemmli buffer, fractionated on a 15% SDS-polyacrylamide gel, and transferred onto nitrocellulose. The membrane was incubated at RT for 3 h in blocking solution (5% non-fat dried milk in 150-mM NaCl, 10-mM Tris pH 8.0, and 0.05% Tween-20), probed at RT for 12 h with anti-BMP5 primary antibodies (anti-PRO 1:667 or anti-MAT 1:172) diluted in blocking solution, and incubated for 1 h at RT in blocking solution containing donkey anti-rabbit-IgG coupled to horseradish peroxidase diluted 1:5000 (anti-PRO) or sheep anti-mouse-IgG coupled to horseradish peroxidase diluted 1:1000 (anti-MAT). Signals on the blot were detected by the ECL system (Amersham).

### *In situ *hybridization

Forelimbs were dissected from E13.5 CD1 embryos, frozen immediately in O.C.T. embedding medium (VWR), and stored at -80°C. Twelve-micrometer cross sections of the humerus at the level of the deltoid tuberosity were collected with a cryostat microtome and were processed, prehybridized, and hybridized with digoxygenin-labeled cRNA probes, as described in previously published protocols [40]. *Bmp *probes were generated by *in vitro *transcription of linearized constructs containing 200- to 500-bp DNA inserts from the pro region or untranslated region of *Bmp *genes as described previously [59]. Descriptions of the washing conditions and the methods used to detect the labeled signal have been published previously [40].

### Isolation of primary osteoblasts and muscle fibroblasts

Primary osteoblast and muscle fibroblast isolates from sex-matched 10-month-old C57Bl/6J and *Bmp5*^*clv *^mice were established. Under microscopy, the humerus was exposed and the surrounding musculature was dissected off the bone. A ~3-mm segment of the humerus containing the deltoid tuberosity (wild type) or the deltoid muscle attachment site (*Bmp5*^*clv*^) was removed. Similarly, a ~5-mm segment of the femur shaft containing the femoral trochanter was dissected from each mouse. The marrow cavity of the bone segments was flushed with ice cold phosphate-buffered saline, and the bone was rinsed in phosphate-buffered saline containing penicillin G (200 U/mL), streptomycin (200 μg/mL), and amphotericin B (0.5 μg/mL). The rinsed bones were minced into small pieces with a bone cutter in 2 mL dissociation medium (21.3-mM Tris pH 7.4, 111.2-mM NaCl, 5.4-mM KCl, 1.3-mM MgCl2, 0.5-mM ZnCl2, and 13-mM glucose supplemented with type II collagenase [5 U/mL], elastase [6.25 U/mL], D-sorbitol [18.22 mg/mL], and chondroitin sulfate [6 mg/mL]) and digested at 37°C for up to 180 min. At 10, 20, and 30 min and every 30 min thereafter, the dissociated cells were removed with the medium and added to 1 volume of FBS (Invitrogen), and a fresh 2 mL of dissociation medium was added to the bone fragments. Cells from the 10–20 min, 30–90 min, and 120–180 min time points were pooled and recovered by centrifugation, resuspended in osteoblast growth medium (αMEM with 10% FBS, 10% horse serum [Invitrogen], 2-mM GlutaMax, and 0.1-mM nonessential amino acids) supplemented with penicillin G (100 U/mL), streptomycin (100 μg/mL), and amphotericin B (0.25 μg/mL), and plated in a 175-cm^2 ^tissue culture flask. The plated cells were allowed to attach for 72 h at 37°C in 5% CO_2_/95% air, and non-adherent cells were rinsed off. Fresh growth medium with antibiotics and antimycotics was replaced every 4 days until the osteoblasts reached confluency (typically ~14 days after initial plating), when they were passaged and maintained similarly afterwards. An aliquot of cells from each flask was plated separately at first passage and assayed for alkaline phosphatase activity, an indicator of osteogenic differentiation, using the AP assay kit (Sigma). Cultures containing fewer than 50% alkaline phosphatase-positive cells were discarded. Antibiotics and antimycotics were omitted from the growth medium after the second passage. Experiments were typically performed on cells from the second and third passages.

A previously described fibroblast isolation protocol [60] was used to harvest primary fibroblasts from the distal third of the deltoid muscle by enzymatic dissociation. The fibroblasts were maintained in DMEM with 15% FBS, 2-mM GlutaMax, and 0.1-mM non-essential amino acids supplemented with penicillin G (100 U/mL), streptomycin (100 μg/mL), and amphotericin B (0.25 μg/mL).

### Mechanical stimulation and indirect immunofluorescence

A total of 5 × 10^4 ^osteoblasts or 1 × 10^4 ^muscle fibroblasts per well were plated on BioFlex 6-well plates with collagen 1-coated flexible membrane bottoms (Flexcell) and allowed to attach overnight. For noggin treatment, fresh medium containing no (control) or recombinant mouse noggin protein (R&D, 0.1–10 μg/mL) was replaced 3 h before mechanical strain. The plates were mounted onto the base plate of a Flexercell FX-4000T system (Flexcell) and left undisturbed for 1 h before initiation of mechanical stimulation to minimize the extraneous strain and fluid stress secondary to handling of the plates.

Cyclic uniform radial strain was applied with -70 kPa negative pressure to produce a 15% maximum elongation of the membranes in square wave cycles of 10 sec strain and 10 sec relaxation at a frequency of 0.05 Hz or 3 cycles per minute.

We visualized the cells using a previously described indirect immunofluorescence protocol [60] with the following modifications. The cells were fixed in ice-cold 50% methanol/50% acetone at -20°C for 20 min; blocked in 10% serum with 0.5% Triton-X (Biorad); and probed with primary antibodies against type I collagen (Chemicon), vimentin (Sigma), or SMAD1/5 (Chemicon) and secondary antibodies coupled to FITC or Cy3 (Sigma). Nuclei were counterstained with propidium iodide (Molecular Probes). The round flexible membrane was cut into equal quadrants and each quadrant was mounted onto a slide. This allowed the angles between the cell axis and the strain field to be measured accurately. Angles were measured from 80–150 cells per condition over 3 independent experiments, and SMAD subcellular localization was determined from 80–150 cells per condition over 3 independent experiments. Images were captured with a Retiga 1300 digital camera (QImaging) attached to a Leica DM IRB microscope and were processed with Northern Eclipse 6.0 software (MVIA). *P *values and statistical significance between the means of each group were determined by the modified Bonferroni's *t *test.

## Authors' contributions

AMH and PCM carried out the genetic sequencing of the *Bmp5*^*clv *^allele. AMH performed the genetic, biochemical, and biomechanical studies on the mutant. DMK and AMH contributed to the conception, design, analysis, and interpretation of the genetic and biochemical analyses. AMH, AJQ, HP, and JH participated in the conception, design, analysis, and interpretation of the biomechanical studies. All authors were involved in the drafting, review, and revision of the manuscript, and have read and agreed to its final content.
